# Effects of High Intensity Circuit Versus Traditional Strength Training on Physiological Responses in Trained Women

**DOI:** 10.1002/ejsc.12298

**Published:** 2025-05-05

**Authors:** Sohee L. Carpenter, Eric R. Helms, Rachel C. Pendakur, Jamie E. Hibbert, Matthew M. Schubert

**Affiliations:** ^1^ Sport Performance Research in New Zealand (SPRINZ), Auckland University of Technology Auckland New Zealand; ^2^ Department of Exercise Science and Health Promotion, Muscle Physiology Laboratory Florida Atlantic University Boca Raton Florida USA; ^3^ SoheeFit Systems LLC San Diego California USA; ^4^ School of Veterinary Medicine University of Wisconsin‐Madison Madison Wisconsin USA; ^5^ Metabolism and Applied Physiology Laboratory California State University San Marcos San Marcos California USA

**Keywords:** circuit training, hypertrophy, resistance, strength, weight lifting, women

## Abstract

This study’s purpose was to compare differences in strength and body composition following high intensity circuit training (HICT) and traditional strength training (TS) in trained women. Fourteen participants (28.5 ± 5.4 years, 160.7 ± 6.1 cm, 65.9 ± 11.7 kg) were randomly assigned to either the HICT group (HICT: *n* = 7) performing two short circuits with 5 min’ rest between each series or the TS group (TS: *n* = 7) performing one exercise at a time, resting 3 min between sets. Both groups trained 3 days per week for 8 weeks [8–15 repetitions, three sets], close to muscular failure. Body composition, arm and thigh muscle girth, skinfold thickness, and 3RM on six different exercises were assessed before and after the intervention. Both groups similarly increased 3RM across all exercises (*p* < 0.001). There was a main effect of time (*p* < 0.001) for both groups on lean body mass indicating an increase in lean mass over time following both protocols, whereas body fat percentage decreased as a factor of time only (*p* = 0.04). There were no significant group or group × time interactions for any other variable. Therefore, both HICT and TS are similarly effective for increasing strength and lean body mass and decreasing body fat percentage in trained women. Those interested in maximizing time efficiency may prefer HICT, as these sessions took much less time to complete. Other variables such as exercise selection and personal preference should also be taken into consideration when choosing training style.

**Summary**

- The result of the present study shows that both HICT and TS are equally effective in increasing strength and LBM, which may result in similar decreases in body fat percentage in trained women when sets are terminated close to failure.
- HICT was able to complete the same amount of work in less time and thus had higher training density compared to TS. HICT had an average completion duration of 50–60 min per session, whereas TS had an average completion duration of 75–85 min per session.
- HICT may be preferable to those interested in maximizing time efficiency, though factors such as exercise selection, equipment availability, personal preference, and ability to sustain a higher work:rest ratio should be taken into consideration.

## 1 Introduction

Resistance training is a form of exercise that utilizes external resistance such as dumbbells, barbells, exercise machines, and body weight to strengthen the body. Resistance training elicits numerous adaptations including reducing fat mass (Wewege et al. 2022) as well as risk of osteoarthritis, osteoporosis, and osteopenia (Going and Laudermilk 2009); increasing muscle mass (Westcott et al. 2009), strength (Grgic et al. 2018), power (Harries et al. 2012; Straight et al. 2016), and bone mineral density (Bocalini et al. 2009; Mosti et al. 2014); decreasing symptoms of depression and anxiety; and improving sleep quality and cognitive abilities (O'Connor et al. 2010) and more.

Circuit training (CT) and traditional strength training (TS) are two dominant forms of resistance training using body weight, free weights, resistance bands, machines, or any combination thereof. CT involves repeating a sequence of exercises one after another with little to no rest between sets. In contrast, TS usually entails performing all sets of one exercise at a time with moderate to long rest periods between sets.

Circuit training is a time‐efficient way to improve muscle mass and strength as well as cardiorespiratory fitness (Brentano et al. 2008; Camargo et al. 2008; Harber et al. 2004). However, CT is typically performed with low loads, not necessarily to failure, and is thus less effective for improving bone mineral density and maximal strength compared to training at closer proximity to muscular failure (Brentano et al. 2008; Harber et al. 2004; Paoli et al. 2010). High intensity CT (HICT) is a form of CT that involves training with higher loads closer to muscular failure and increases strength, lean mass, and bone mineral density (Romero‐Arenas et al. 2013).

On the other hand, TS typically utilizes heavier loads with moderate to long rest periods between sets compared to CT (Kraemer and Ratamess 2004). TS significantly improves bone health (Bocalini et al. 2009; Menkes et al. 1993) as well as muscle mass (Benito et al. 2020; Blazevich et al. 2007; Schoenfeld et al. 2017; Suetta et al. 2008) and strength (Alcaraz et al. 2008). Some drawbacks of TS include the time required to complete a workout session, moderate cardiovascular health benefits compared to other forms of training (e.g., CT and aerobic training), and minimal body fat loss (Alcaraz et al. 2011).

To date, there is limited research comparing the physiological effects of HICT compared to TS, and of the research that does exist, the vast majority is conducted in men. A 2011 study in trained men found that 8 weeks of HICT elicited similar gains in strength and muscle mass compared to TS (Alcaraz et al. 2011). Another study in an elderly population also found that HICT was as effective as TS at increasing strength, muscle mass, and bone mineral density and more effective at inducing cardiovascular adaptations and decreasing fat mass (Romero‐Arenas et al. 2013). To our knowledge, however, there are no studies specifically comparing HICT to TS in trained premenopausal women, and the lack of research on this topic means that the existing findings in men cannot necessarily and confidently be applied to women. As women’s participation rates in resistance training remain at a mere 25.8% in the United States (Bennie et al. 2018), this speaks to the need for more resources devoted to and knowledge of resistance training in women to increase adherence rates overall.

Therefore, the purpose of this study was to determine the effects of HICT versus TS on strength, muscle girth, and body composition in trained women. It was hypothesized that HICT would not be significantly different from TS for increasing strength, muscle girth, and LBM but would be more effective for decreasing body fat compared to TS, similarly to previous research in trained men (Alcaraz et al. 2011).

## 2 Methods

### 2.1 Experimental Approach to the Problem

To compare the effects of HICT versus TS on strength, muscle girth, and body composition in women, a longitudinal randomized parallel group trial was conducted. Participants were recruited using fliers posted across the [SCHOOL NAME] campus and on various social media platforms. The independent variable was the‘training condition’—namely, HICT or TS. Dependent variables included 3‐repetition maximum strength (3RM) strength, muscle girth, skinfold thickness, and body composition.

### 2.2 Subjects

Due to the likelihood that logistics would constrain our sample size, rather than relying on an a priori sample size calculation, we recruited as many participants as we could between September 2022 and February 2023. A total of 20 women volunteered for the study, and by the end, 14 participants completed the study (HICT = 7; TS = 7). Inclusion criteria were English‐speaking women between the ages of 18 and 40 years old, trained (≥ 6 months of resistance training experience with a minimum training frequency of two times per week), healthy, and free from musculoskeletal injury. Participants agreed not to take ergogenic aids, supplements, or medications that might affect resistance training performance and were asked to maintain their accustomed diets throughout the duration of the study. Participants were informed of the benefits and risks of the investigation prior to signing an informed consent form to participate in the study. They were free to withdraw from the study at any time. Participant characteristics are summarized in Table 1. A minimum of 80% attendance was required for a participant to complete the study. One participant withdrew due to an injury sustained outside the research, three withdrew as a result of missing too many sessions, and two withdrew due to mild discomfort incurred during the training protocol which prevented participation at the required intensity and/or frequency. The methods and procedures used in this study were approved by the Auckland University of Technology Ethics Committee, AUTEC Reference No. 22/16. The study was also approved by the California State University San Marcos Institutional Review Board (IRB) #1925058‐1.

**TABLE 1:** Participant characteristics at baseline (mean ± SD).

	HICT	TS	Effect size (Cohen’s d)	95% CI for group	*p*
Age (years)	30.10 ± 4.65	26.94 ± 5.92	0.59	−0.49 to 1.66	0.29
Height (cm)	160.34 ± 7.17	161.16 ± 5.27	−0.13	−1.18 to 0.92	0.81
Weight (kg)	66.74 ± 5.05	65.14 ± 16.40	0.25	−0.92 to 1.18	0.81
Body fat percentage (%)	33.39 ± 7.28	34.50 ± 7.80	−0.15	−1.19 to 0.91	0.79
3RM leg press (kg)	112.75 ± 29.01	79.05 ± 42.30	−0.93	−0.20 to 2.02	0.11
3RM flat DB bench press (kg)	29.20 ± 5.82	25.30 ± 4.24	0.76	−0.34 to 1.84	0.18
3RM trap bar deadlift (kg)	72.90 ± 15.24	65.12 ± 19.28	0.45	−0.62 to 1.50	0.42
3RM lat pulldown (kg)	40.18 ± 5.34	37.58 ± 6.64	0.43	−0.64 to 1.48	0.44
3RM hip thrust (kg)	113.07 ± 33.73	107.57 ± 16.87	0.21	−0.85 to 1.25	0.71
3RM standing DB shoulder press (kg)	22.12 ± 3.36	19.12 ± 4.32	0.78	−0.33 to 1.86	0.17

### 2.3 Procedures

During the pre‐intervention phase, participants first took part in a familiarization session. Following a 5‐min treadmill walk at a brisk pace, they completed a dynamic warm‐up consisting of the following exercises for 10 reps each: bent over thoracic spine rotation, body weight windmill, quad stretch to hamstring sweep, lateral squat, and dynamic shoulder circle. Next, they performed one set of each exercise (leg press, flat DB bench press, trap bar deadlift, lat pulldown, hip thrust, and standing DB shoulder press) at a 5–7 rating (0–10 Borg scale) of perceived exertion (RPE) within an 8–15 target rep range. The purpose of this phase was to ensure that the participants were familiar with the movements and allow the researcher to check the participants’ exercise technique, making adjustments as needed. The second session, which took place 48–72 h later, consisted of 3RM testing for each exercise. The third session consisted of the body composition assessment and took place 48–72 h following the previous sessions. The participants were then randomly assigned to the HICT or TS group for the duration of the training intervention. Exercise descriptions were as follows:

*Leg press:* Sit down in the machine and position the feet on the foot pad roughly shoulder‐width apart. Extend the legs until the knees lock out, and then lower the load under control until the legs have reached roughly a 45° angle.

*Flat DB bench press:* Holding a dumbbell in each hand, lie back on a flat bench. With the arms positioned on either side of the torso, press the dumbbells up until the elbows lock out and then lower under control.

*Trap bar deadlift:* Stand inside the trap bar and hold the handles with both hands. While maintaining a relatively neutral spine and a soft bend in the knees, pull the load off the floor by thinking of pushing into the ground. Extend the hips and stand tall, then reverse the motion and lower the load back to the floor under control.

*Lat pulldown:* Grab the bar with an approximately shoulder‐width overhand grip. While maintaining torso upright, pull the elbows down toward the hips and bring the bar to the chest, then slowly return to the start position.

*Hip thrust:* Sit with a barbell across the hips and the upper back against a bench 12–14″ in height. Push through the heels and extend the hips, keeping the head forward and chin tucked. Squeeze the glutes at the top of the repetition and then lower back down under control.

*Standing DB shoulder press:* Holding a dumbbell in each hand by the shoulders, press the dumbbells overhead until the elbows lock out, and then lower the dumbbells under control.

Training consisted of three weekly sessions performed on nonconsecutive days for a total of 8 weeks. Initial loads started at approximately 65% of their 3RM, rounded down to the nearest 1.25 kg (kg). From there, participants performed sets consisting of 8–15 repetitions at an 8 or 9 on the Borg CR10 RPE scale. Loads were increased or decreased in subsequent sets as needed to maintain the appropriate perceived exertion (RPE 8–9) and target repetition range (8–15 per set).

The HICT group completed exercises “vertically” (one set completed per exercise before moving onto the next exercise) in two separate three‐exercise series or circuits with 35 s of rest between sets (enough time to transition safely between exercises) and a 5‐min break between each series. Participants would move from one exercise to the next (e.g., one set of leg press, one set of flat DB bench press, and then one set of trap bar deadlift), repeating the series until three sets of each exercise had been completed. The first circuit consisted of leg press, flat DB bench press, and trap bar deadlift; the second circuit consisted of lat pulldown, hip thrust, and standing DB shoulder press.

Meanwhile, the TS group completed all sets of one exercise at a time, resting 3 min between sets, before moving onto the next exercise, with up to a 5‐min break after the first three exercises. In other words, those in the TS group would complete the first set of leg press, rest 3 min, then a second set of leg press, rest 3 min, and then a final set of leg press before moving onto the second exercise, the flat DB bench press, and so on.

**FIGURE 1:**
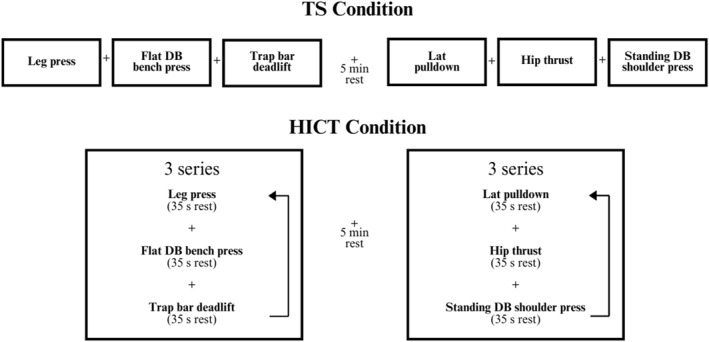
Experimental design: TS performed three sets of each exercise one at a time with a 3‐min rest between sets and a 5‐min rest allowed after the third exercise; HICT performed three series of two circuits with a 5‐min rest.

Both groups thus completed the same exercises for the same repetition range and number of sets, and they differed only in the order in which the exercises were executed as well as the rest taken between sets. The warm‐up, RPE, and rep ranges were the same between groups. Participants were supervised by at least one experienced trainer at every session to ensure the exercise technique met the required range of motion standards, each set was terminated close to failure, and rest periods were strictly enforced. See Figure 1.

**FIGURE 2:**
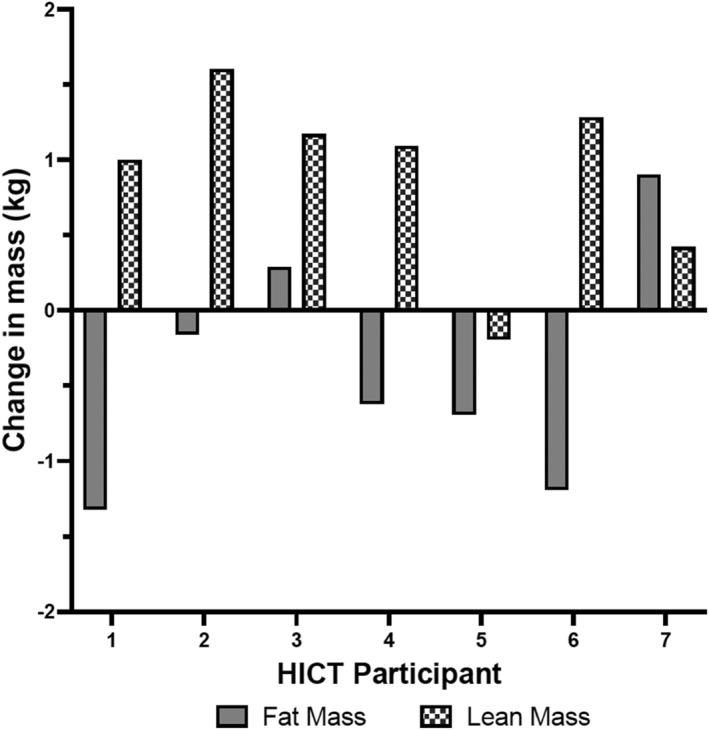
Individual changes in LBM and FM in the HICT group.

Participants were asked to maintain their usual eating habits and to not ingest any supplements that may affect their training performance (Figure 2). Participants were required to attend a minimum of 20 of the 24 training sessions to complete the study.

*Strength:* 3RM strength of each exercise was tested prior to beginning and after completion of the training protocol. These testing sessions were not included in the 24 training sessions. Following a 5‐min steady‐state cardiovascular warm‐up consisting of brisk walking on a treadmill and the 5‐min dynamic warm‐up described in the first paragraph of the procedures, participants completed two sets of 5–8 repetitions of each exercise at a submaximal load with 2 min of rest between sets. Then, an initial load was lifted for three reps that was within an estimated 50%–70% of the participants’ capacity. The selection of this load was informed by the prior experience of the participant and gauged by the investigator who is an experienced trainer. After a load was successfully lifted, the participants were allowed to rest 3–5 min before attempting another lift with an additional load increased by 1.25–20 kg (depending on participant strength and the difficulty of the prior set) for three repetitions. Participants were allowed up to five total 3RM attempts. If the final attempt was one to two repetitions, this load was used to calculate their 3RM using the NSCA training load chart (Landers 1984).

*Muscle girth and skinfold thickness*: Girth and skinfold measurements were performed on the right limbs at the midpoint between the humeral greater tuberosity and lateral epicondyle for the arm and the midpoint between the greater trochanter and lateral epicondyle of the femur for the leg. Muscle girth measurements were obtained with a steel tape measure, and skinfold measurements were performed in a rotational order using a metal skinfold caliper (Lange, Santa Cruz, CA) at the same sites. All measurements were performed by the same researcher in triplicate to eliminate inter‐rater variability. The mean of the two closest values was recorded for each site. Measurements were taken at pre‐ and post‐intervention.

*Body composition:* Height was measured using a stadiometer, and body mass was measured using a balance beam scale (Health‐o‐Meter; Creative Health Products, Ann Arbor, MI). Participants had their body composition measured via dual‐energy X‐ray absorptiometry (DXA, Lunar Prodigy, GE Corp, Madison, WI) pre‐ and post‐intervention at the [SCHOOL NAME] kinesiology lab. The DXA coefficient of variation in the [SCHOOL NAME] kinesiology lab is 0.8%–5.9%, and intraclass correlation (ICC) reliability is 0.996 for percent body fat, 0.975 for fat mass (FM), and 0.994 for fat‐free mass (Schubert et al. 2019). Body composition measurements were conducted by the same technician using the lab’s established protocols for best and standardized practice. Participants were asked to refrain from alcohol, caffeine, and moderate/vigorous exercise for at least 14 h before each visit and to abstain from food 12 h before testing. Even so, not all participants were able to adhere fully to the standardized conditions (namely, abstaining from food 12 h before testing), and timing was not always consistent between pre‐ and post‐intervention assessments due to scheduling logistics. Although the data of these participants could have been excluded, doing so would have yielded an even smaller sample size, and so the decision was made to retain data even when standardization was not perfectly followed and to acknowledge this limitation. EnCore version 17 (GE Corporation, Madison, WI, USA) was used to calculate percent body fat, FM, and lean body mass. The software further differentiated bone mass from lean mass.

### 2.4 Statistical Analyses

All data are reported as means ± standard deviations. A two‐way analysis of variance (ANOVA) with repeated measures (group × time) was used to compare differences in pre‐ and post‐intervention 3RM strength, lean body mass, and body composition between groups. Effect size statistics were used to determine the magnitude of differences within and between the two groups and were defined as small, medium, and large represented by Cohen’s *d* of greater than 0.2, 0.5, and 0.8, respectively. Confidence intervals (95% CI) were presented where appropriate. Significance was accepted when *p* < 0.05. All analyses were completed using JASP (v 0.17.2.1).

## 3 Results

The study was based on 14 participants who responded to the study invitation and completed the study with a minimum attendance of 80%. At the end of the study, the number of participants in each group were as follows: HICT, *n* = 7; TS, *n* = 7. Out of a total of 24 sessions, attendance was 21.00 ± 1.41 for HICT and 21.14 ± 1.46 for TS, with no significant difference in attendance between groups (*p* = 0.86). The average number of repetitions performed per set throughout the training intervention was 9.97 ± 0.56 for HICT and 9.45 ± 0.97 for TS, with no significant difference between groups (*p* = 0.24).

Pretraining characteristics of subjects in each training group are presented in Table 1. No significant pre‐intervention differences in any participant characteristics or 3RM strength measures were found between HICT and TS. Average loads for all exercises and total volume between groups are displayed in Tables 2 and 3, respectively.

**TABLE 2:** Average load for each exercise throughout training intervention.

Exercise (kg)	HICT	TS	Effect size (Cohen’s d)	95% CI for group	*p*
Leg press	89.61 ± 23.65	83.94 ± 35.79	0.19	−0.87 to 1.23	0.73
Flat DB bench press	25.70 ± 4.90	24.15 ± 3.54	0.36	−0.70 to 1.41	0.51
Trap bar deadlift	61.60 ± 8.50	63.54 ± 14.20	−0.16	−1.21 to 0.89	0.76
Lat pulldown	32.45 ± 4.28	33.12 ± 4.63	−0.15	−1.20 to 0.90	0.78
Hip thrust	88.44 ± 17.86	93.16 ± 13.52	−0.30	−1.35 to 0.76	0.59
Standing DB shoulder press	16.69 ± 3.30	17.36 ± 3.07	−0.21	−1.26 to 0.84	0.70

**TABLE 3:** Training volume for each exercise throughout training intervention.

Exercise (kg)	HICT	TS	% Difference	Effect size (Cohen’s d)	95% CI for group	*p*
Leg press	59,780.51 ± 15,539.95	53,642.11 ± 25,640.36	−10.27	0.29	−0.77 to 1.34	0.60
Flat DB bench press	16,773.51 ± 5099.25	14,359.69 ± 2178.05	−14.39	0.62	−0.47 to 1.68	0.27
Trap bar deadlift	33,666.89 ± 14,576.81	35,454.63 ± 7907.59	5.31	−0.15	−1.20 to 0.90	0.78
Lat pulldown	19,681.35 ± 3150.00	19,419.05 ± 2519.26	−1.33	0.09	−0.96 to 1.14	0.87
Hip thrust	55,642.13 ± 13,585.51	57,206.70 ± 8965.06	2.81	−0.14	−1.18 to 0.92	0.80
Standing DB shoulder press	9983.66 ± 2075.51	9862.75 ± 1585.28	−1.21	0.07	−0.98 to 1.11	0.91
Total	195,528.06 ± 37,460.88	189,944.92 ± 43,539.06	−2.86	0.14	−0.91 to 1.18	0.80

### 3.1 Strength

The results of all strength changes pre‐ and post‐intervention are displayed in Table 4. A statistically significant main effect of time indicated an increase in strength for every exercise (*p* < 0.001; 84.97 ≥ *F* ≤ 232.83); however, the main effects for group and interactions for all exercises were not statistically significant.

**TABLE 4:** 3RM strength.

Exercise (kg)	Pre‐intervention	Post‐intervention	% Change	Effect size (Cohen’s d)	95% CI for group	*p* ^a^	*F* ^a^
Leg press							
HICT	112.75 ± 29.01	189.53 ± 22.49^b^	68.10	2.96	−0.44 to 1.91	0.46	0.59
TS	79.05 ± 42.30	163.94 ± 58.63^b^	107.39	1.66			
Flat DB bench press							
HICT	29.20 ± 5.82	35.58 ± 6.06^b^	21.85	1.07	−0.60 to 1.69	0.24	1.56
TS	25.30 ± 4.24	33.70 ± 4.90^b^	33.20	1.83			
Trap bar deadlift							
HICT	72.90 ± 15.24	94.28 ± 12.69^b^	29.33	1.52	−0.83 to 1.47	0.29	1.21
TS	65.12 ± 19.28	90.72 ± 22.49^b^	39.31	1.22			
Lat pulldown							
HICT	40.18 ± 5.34	51.70 ± 8.30^b^	28.67	1.65	−0.90 to 1.36	0.32	1.07
TS	37.58 ± 6.64	51.06 ± 7.26^b^	35.87	1.94			
Hip thrust							
HICT	72.90 ± 15.24	154.54 ± 25.37^b^	36.68	1.39	−1.07 to 1.17	0.27	1.33
TS	65.12 ± 19.28	157.46 ± 18.51^b^	46.38	2.82			
Standing DB shoulder press							
HICT	22.12 ± 3.36	26.84 ± 3.26^b^	21.34	1.43	−0.56 to 1.79	0.08	3.68
TS	19.02 ± 4.32	25.34 ± 3.60^b^	33.23	1.56			

^a^ Values for group × time interaction.

^b^ Significantly different from pre‐intervention.

### 3.2 Muscle Girth and Skinfold Thickness

Muscle girth and skinfold thickness results are displayed in Table 5. There were no statistically significant main effects of time (0.31 ≥ *p* ≤ 0.52; 0.43 ≥ *F* ≤ 0.97), group (0.21 ≥ *p* ≤ 0.88; 0.03 ≥ *F* ≤ 1.75), or interactions (0.30 ≥ *p* ≤ 0.70; 0.15 ≥ *F* ≤ 1.16) for any sites examined (Figure 1).

**TABLE 5:** Muscle girths and skinfold thicknesses.

	Pre‐intervention	Post‐intervention	% Change	Effect size (Cohen’s d)	95% CI for group	*p* ^a^	*F* ^a^
Girth—Right arm (cm)							
HICT	26.68 ± 4.14	26.08 ± 2.90	−2.25	0.17	−1.07 to 1.34	0.36	0.92
TS	26.08 ± 3.93	26.05 ± 3.99	−0.12	0.01			
Girth—Right leg (cm)							
HICT	56.26 ± 5.01	54.61 ± 3.70	−2.93	0.37	−0.95 to 1.28	0.35	0.94
TS	54.27 ± 6.14	54.67 ± 7.83	0.74	0.06			
Skinfold—Right arm (mm)							
HICT	22.43 ± 3.92	22.84 ± 5.00	1.83	0.09	−0.87 to 1.42	0.70	0.16
TS	20.36 ± 7.70	21.36 ± 8.43	4.91	0.12			
Skinfold—Right leg (mm)							
HICT	29.71 ± 5.58	28.89 ± 5.04	−2.76	0.15	−0.49 to 1.88	0.70	0.15
TS	25.14 ± 7.39	24.79 ± 6.70	−1.39	0.05			

^a^ Values for group × time interaction.

### 3.3 Body Composition

**FIGURE 3:**
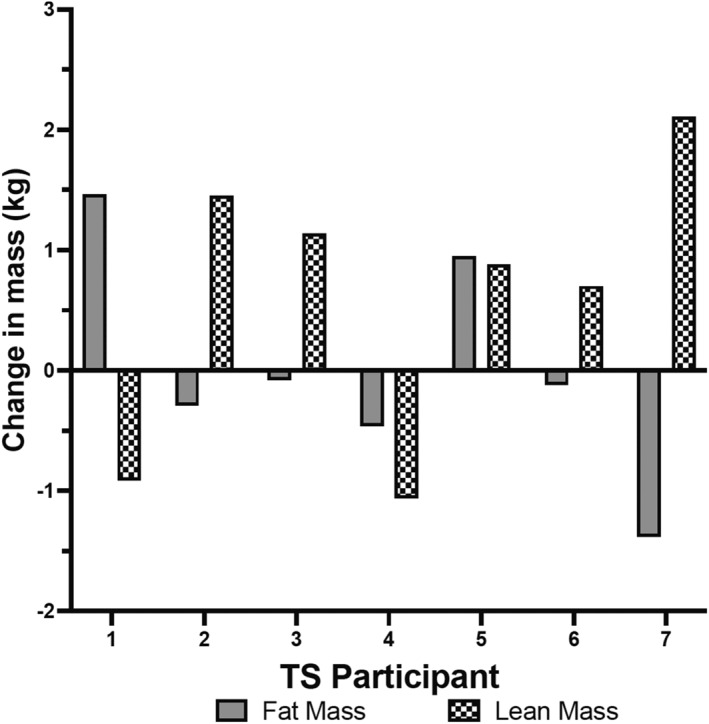
Individual changes in LBM and FM in the TS group.

Body composition results are displayed in Table 6. There were no statistically significant (*p* = 0.84, *F* = 0.04) main effects of time, group (*p* = 0.82, *F* = 0.05), or interactions (*p* = 0.70, *F* = 0.16) for body mass. For both interventions, there was a statistically significant main effect of time indicating an increase in LBM (*p* = 0.01, *F* = 9.23) and a decrease in body fat percentage (*p* = 0.04, *F* = 5.33). However, the main effect for the group (*p* = 0.66, *F* = 0.29) and interaction (*p* = 0.60, *F* = 0.34) was not statistically significant for LBM, and similarly, the main effect of the group (*p* = 0.75, *F* = 0.11) and interaction (*p* = 0.53, *F* = 0.41) was not statistically significant for body fat percentage. There were no statistically significant main effects of time (*p* = 0.42, *F* = 0.69), group (*p* = 0.96, *F* = 0.00), or interactions (*p* = 0.40, *F* = 0.78) for FM. There were no statistically significant main effects of time (0.23 ≥ *p* ≤ 0.95; 0.14 ≥ *F* ≤ 0.88), group (0.23 ≥ *p* ≤ 0.85; 0.04 ≥ *F* ≤ 1.54), or interactions (0.32 ≥ *p* ≤ 0.80; 0.07 ≥ *F* ≤ 1.07) for arm, leg, and trunk LBM; similarly, there were no statistically significant main effects of time (0.14 ≥ *p* ≤ 0.88; 0.03 ≥ *F* ≤ 2,54), group (0.64 ≥ *p* ≤ 0.86; 0.03 ≥ *F* ≤ 0.23), or interactions (0.50 ≥ *p* ≤ 0.94; 0.01 ≥ *F* ≤ 0.47) for arm, leg, and trunk FM. Individual changes in LBM and FM for HICT and TS are displayed in Figure 3, respectively.

**TABLE 6:** Body composition.

	Pre‐intervention	Post‐intervention	% Change	Effect size (Cohen’s d)	95% CI for group	*p* ^a^	*F* ^a^
Body mass (kg)							
HICT	66.74 ± 5.05	66.68 ± 5.44	−0.09	0.01	−1.04 to 1.29	0.70	0.16
TS	65.14 ± 16.40	65.31 ± 15.96	0.26	0.01			
Body fat (%)							
HICT	33.39 ± 7.28	32.40 ± 6.75	−2.96	0.14	−1.34 to 0.99	0.53	0.41
TS	34.50 ± 7.80	33.94 ± 8.57	1.56	0.07			
LBM total (kg)							
HICT	41.12 ± 4.64	42.03 ± 4.25^b^	2.21	0.20	−0.88 to 1.46	0.57	0.34
TS	39.85 ± 5.77	40.47 ± 4.85	1.56	0.12			
LBM arms (kg)							
HICT	4.06 ± 0.58	4.04 ± 0.58	−0.49	0.03	−0.64 to 1.57	0.80	0.07
TS	3.74 ± 0.75	3.78 ± 0.53	1.07	0.06			
LBM legs (kg)							
HICT	14.60 ± 1.66	15.09 ± 1.57	3.36	0.30	−0.50 to 1.71	0.32	1.07
TS	13.66 ± 2.31	13.10 ± 3.55	−4.10	0.19			
LBM trunk (kg)							
HICT	19.65 ± 2.65	20.08 ± 2.62	2.19	0.17	−1.05 to 1.25	0.60	0.28
TS	19.49 ± 3.29	19.67 ± 2.52	0.92	0.06			
FM total (kg)							
HICT	22.98 ± 5.14	22.58 ± 5.34	−1.74	0.08	−1.14 to 1.19	0.40	0.78
TS	22.55 ± 11.21	22.56 ± 11.55	0.04	< 0.001			
FM arms (kg)							
HICT	2.27 ± 0.35	2.16 ± 0.49	−4.85	0.26	−0.88 to 1.38	0.94	0.01
TS	2.14 ± 0.59	2.02 ± 0.72	−5.61	0.18			
FM legs (kg)							
HICT	8.76 ± 1.54	8.64 ± 1.74	−1.37	0.07	−0.94 to 1.40	0.59	0.31
TS	8.01 ± 3.63	8.04 ± 4.01	0.37	0.01			
FM trunk (kg)							
HICT	11.16 ± 3.96	10.97 ± 3.98	−1.70	0.05	−1.26, 1.07	0.50	0.47
TS	11.56 ± 7.28	11.67 ± 7.15	0.95	0.02			

^a^ Values for group × time interaction.

^b^ Significantly different from pre‐intervention.

## 4 Discussion

To the authors’ knowledge, this was the first study to investigate the physiological effects of HICT versus TS in trained premenopausal women. The primary finding was that both HICT and TS yielded similar increases in strength and LBM and a decrease in body fat percentage, with no statistically significant change in muscle girth, skinfold thickness, body mass, or FM. The hypothesis that HICT’s impact on strength, muscle girth, and LBM would not significantly differ from TS’s was supported, but the hypothesis that only HICT would experience a reduction in body fat percentage was not.

With regards to the changes in LBM and FM, individual data displayed in Figures 2 and 3 indicate that several participants experienced noteworthy increases in LBM and decreases in FM. Specifically, 4 HICT participants gained 1 kg or more LBM, and 3 TS participants gained 1 kg or more LBM, with one TS participant gaining 1.45 kg LBM and losing 0.29 kg FM and another TS participant gaining 2.11 kg and dropping 1.35 kg FM. A total of eight out of the 14 total participants experienced simultaneous muscle gain and fat loss, a relatively common phenomenon known as body recomposition (Barakat et al. 2020). This is especially marked given these changes occurred within 8 weeks in the absence of any dietary intervention.

Our results demonstrate that even when rest periods are shorter, as in the HICT group, similar strength and LBM gains can be achieved as long as sets are terminated close to muscular failure. This outcome may also be due in part to the exercise order in the HICT group, as rather than straight sets, the circuit nature allowed the specific muscles which were trained on a given exercise to rest during subsequent exercises which trained different musculatures.

These results corroborate the findings of Brentano et al. (2008) which found no significant differences in isometric strength, upper limb dynamic strength, and lower limb dynamic strength between CT and TS after 24 weeks of training in untrained postmenopausal women with bone loss. It should be noted that in that particular study, those in the CT group trained at lower intensities, thus their style of training could not be classified as HICT, yet strength increases were still observed. The results are also in line with that of Romero‐Arenas et al. (2013), which similarly found that HICT was as effective as TS in increasing strength and muscle mass in an elderly population.

To the authors’ knowledge, there are three prior studies to date specifically examining HICT in women. Two studies found that HICT significantly decreased body fat percentage while increasing strength (Lee et al. 2021; Mehmood et al. 2022), whereas one found that HICT improved strength but did not change body composition (Ludin et al. 2015). However, in all of these studies, the exercises performed by participants in the HICT condition consisted primarily of body weight exercises (e.g., jumping jacks, sit‐ups, squats, and planks) rather than externally loaded gym‐based lifts. Although working sets were completed with high perceived exertion in these studies, unlike in the present study, it was not also specified that they were terminated close to muscular failure, and no prior study directly compared HICT to TS. Still, these findings suggest that it is possible for HICT to significantly increase strength and possibly reduce body fat percentage even while not pushing close to muscular failure and without using external resistance.

Similar to our findings, Alcaraz et al. (2008) found that HICT was equally as effective at TS in improving bench press and half squat 1RM after 8 weeks of training in trained men. The HICT group significantly decreased their body fat percentage by 1.5%, whereas the TS group experienced a nonsignificant decrease of 1.1%, and both groups significantly increased LBM. Further, Paoli and colleagues found that HICT was more effective than low‐intensity CT and endurance training in reducing body mass, FM, and increasing strength in overweight middle‐aged men (Paoli et al. 2010).

Previous research examining CT at lower loads not close to muscular failure in comparison to TS reported superior results following TS for strength and muscle mass increases (Campos et al. 2002). Additionally, the strength and LBM gains occurred in trained individuals with a consistent history of lifting weights in whom additional gains can be slower (Alway et al. 1988). Thus, the ability of HICT to improve strength and muscle mass in those with training experience is noteworthy for populations seeking a more time‐efficient way to exercise. With standardized rest periods making up the majority of the time spent in each session, we had an average completion duration of 50–60 min in HICT and 75–85 min in TS. Thus, HICT was able to complete the same amount of work in less time, meaning the training density of HICT was much greater, with a higher work:rest ratio. However, higher and lower work:rest ratios will likely be perceived differently.

There are other time‐saving strategies available for those seeking strength and body composition alterations besides CT. Specifically, antagonist paired sets, in which exercises that train opposing muscle groups are paired together, which help reduce training time without compromising the training volume and is equally as effective at TS at increasing 1RM strength (Iversen et al. 2021; Robbins et al. 2009). Other advanced time‐saving strategies that seem not to compromise adaptation include drop‐set training in which a traditional set is performed, then immediately followed by another set (or multiple sets) with reduced load (Schoenfeld and Grgic 2018) and the rest‐pause method in which sets are broken up into smaller sets with short breaks in between, thus allowing for maintenance of high loads (Tufano et al. 2017).

Caution should be taken when interpretating the body composition results of this study. Although participants were asked not to make any dietary changes throughout the duration of the training intervention, their diets were not standardized, no restrictions were placed on them, nor were specific nutrition strategies recommended. Rather, pre‐intervention 24‐h food recalls were collected to increase dietary control by raising awareness of participants’ diets and to encourage them to be consistent with their diets throughout the study. However, no mid‐ or post‐intervention diet log was collected, and thus there was no way to confirm whether or not participants maintained the same diet throughout. Future research should either closely monitor dietary intake or examine the effects of dietary interventions implemented concurrently alongside the training interventions to better understand the impact of diet on the outcome variables. For example, it is possible that a protein intake of 1.6 g/kg/day or higher coupled with resistance training can enhance muscle and strength gain (Nunes et al. 2022). Even so, the findings from this study are meaningful in that both HICT and TS observed significant physiological changes despite not controlling for diet, suggesting that modality is effective for increasing strength and LBM in trained women.

Several other limitations arose during the data collection process that must be addressed. Of primary concern was the small sample size. Given the duration of time during which data collection took place as well as the total number of visits, participant interest and availability were limited. Further, although a concerted effort was made to standardize body composition testing conditions pre‐ and post‐intervention, five participants did not remain fasted in the 12 h leading up to their DXA scans, and the timing of their assessments was not consistent from pre‐ to post‐intervention (i.e., some individuals had their assessments in the morning pre‐intervention but late afternoon post‐intervention, or vice versa) due to scheduling logistics. Thus, their body composition results may have contained additional errors. Finally, caution should be taken when extrapolating the findings of this study to a training plan of longer duration or to men, individuals in different age groups, those with different levels of training experience, or those performing a program with different training variables.

## 5 Practical Application

The results of this study suggest that both HICT and TS are viable methods to induce strength and LBM increases and decrease body fat percentage in trained women when sets are terminated close to failure. Notably, there was no significant difference between groups for total training volume or average load, thus indicating that HICT and TS result in similar work, but with less time in HICT. Those who have time constraints to consistent exercise may find HICT more useful, as shorter workouts may be realistic for their busy schedules. These findings also have implications for practitioners designing training programs who may recommend HICT to clients who are busy or would like time‐efficient workouts. When deciding whether to adopt HICT, TS, or a combination of both training styles and personal preference, the ability to sustain a higher work:rest ratio and time constraints can be used as guiding factors because both training styles result in increased strength and LBM and decreased body fat percentage.

Future research should compare perceived exertion, enjoyment, and perceived difficulty between HICT and TS to better ascertain which modality an individual should employ. Additionally, in order to verify that accustomed dietary habits are being maintained throughout the study, participants should keep dietary records. Dietary logs should be assessed pre‐, mid‐, and post‐intervention and analyzed for total calorie intake and protein, carbohydrate, and fat composition using computer software to confirm no confounding effects occur.

## Conflicts of Interest

The authors declare no conflicts of interest.
